# Blood flow-restricted resistance training modulates miRNAs to improve early hypertensive cardiac function

**DOI:** 10.1371/journal.pone.0333027

**Published:** 2025-09-25

**Authors:** Zhaowen Tan, Hao Zhu, Yan Zhao, Yuchan Zheng, Jie Zhang

**Affiliations:** 1 College of Physical Education, Nanjing Xiaozhuang University, Nanjing, China; 2 College of Sports Science, Nanjing Normal University, Nanjing, China; 3 Nanjing Sport Institute, Nanjing, China; Penn State Health Milton S Hershey Medical Center, UNITED STATES OF AMERICA

## Abstract

**Objective:**

The aim of this study was to explore the differentially expressed miRNAs in the hearts of rats protected from early spontaneous hypertension by blood flow-restricted resistance training and to elucidate the effects of blood flow-restricted resistance training on the expression of these genes.

**Methods:**

Four-week-old SHRs and WKY rats were used and randomly divided into five groups: the normal group (WKY), SHR control group (SHR-SED), high-intensity resistance training group (HIRT), medium-intensity resistance training group (MIRT), and blood flow-restricted medium-intensity resistance training group (BFRT). During the experiment, the body weight, cardiac function and hemodynamic parameters of the rats were measured. After training, total RNA was extracted from the left ventricular myocardium of rats in the SHR-SED group and the BFRT group, miRNAs were sequenced, followed by GO enrichment and KEGG pathway analyses, and the differentially expressed miRNAs were subsequently validated via qRT‒PCR.

**Results:**

1) Hemodynamic tests revealed that the blood pressure of SHRs in the BFRT decreased significantly and that the blood pressure level of SHRs in the BFRT decreased more significantly than that of the simple resistance training groups did (P < 0.05). 2) Cardiac function tests revealed that the EF, FS, and MV E/A of SHRs in the BFRT significantly increased, whereas the HR, IVSd, IVSs, LVIDd, LIVDs, LVPWd, LVPWs and LV mass significantly decreased (P < 0.05). 3) Transcriptome sequencing revealed 9 differentially expressed miRNAs in the BFRT group compared with the SHR-SED group (2 miRNAs were significantly upregulated, and 7 miRNAs were significantly downregulated), with P < 0.05 and |log2FoldChange| ≥ 1 used as the criteria for differential significance. The most prominent differentially expressed miRNA was miR-200b-3p (P = 0.00, |log2FoldChange| = 2.45). 4) The miRNA validation results revealed that BFRT significantly reduced the expression of miR-200a-3p, miR-200b-3p, miR-342-3p, miR-350, miR-429, miR-1249, miR-1949 in SHR myocardium, and increased the expression of miR-31a-5p and miR-224-5p (P < 0.01).

**Conclusion:**

Eight weeks of blood flow-restricted medium-intensity resistance training could lower SHR blood pressure, and it might also improve early SHR cardiac function by regulating the expression of miR-224-5p, miR-31a-5p, miR-200b-3p, miR-200a-3p, miR-342-3p, miR-429, miR-1949, miR-1249, and miR-350, with the differential expression of miR-200b-3p being particularly significant.

## 1. Introduction

Hypertension, a high-risk factor for cardiovascular disease, often leads to cardiac dysfunction or organic damage, affects approximately 1 billion people worldwide, and this number continues to rise [1]. A particular concern is that hypertension poses a significant threat to the heart in its early stages, as evidenced by myocardial hypertrophy, fibrosis, and reduced coronary collateral circulation, which are pathological changes that severely impair normal cardiac function [2]. Exercise therapy, as an important therapeutic tool in the nonpharmacological treatment of hypertension, is able to lower blood pressure through multisystemic and nontraditional mechanisms (such as improving cardiovascular health to lower blood pressure and reducing sympathetic overactivation to lower blood pressure), which in turn reduces the risk of cardiovascular mortality [3]. Among them, blood flow-restricted resistance training, as an emerging alternative high-intensity resistance training method, has emerged in the treatment of hypertension in recent years and has demonstrated superior blood pressure lowering effects compared with traditional moderate- and high-intensity resistance training [4,5], which undoubtedly provides hypertensive patients with another effective exercise treatment option [6]. Moreover, some studies have shown that blood flow-restricted resistance training may promote the repair of endothelial cell function and increase the expression of angiogenic factors through the multiple effects of metabolic stress, hypoxia, extrinsic mechanical forces, and laminar shear stress, thus facilitating positive intervention in the pathological factors associated with cardiovascular dysfunction [7]. However, despite the increasing number of studies on blood flow-restricted resistance training and the finding that blood flow-restricted resistance training can provide more benefits than simple resistance training in early hypertension heart protection [8], there is still insufficient research on its efficacy and mechanisms in hypertension heart damage, and more in-depth research in animal models is urgently needed.

In this context, miRNAs, as hotspots for mechanistic studies, have received increasing attention for their key regulatory roles in cell proliferation, differentiation, apoptosis, tumor transformation, metabolism, and other cellular processes [9]. Especially in the field of hypertensive cardiovascular biology, miRNAs are involved in the regulation of almost all important aspects and have become key regulators of hypertensive cardiovascular function [10]. miRNAs such as miR-143/145, miR-21, miR-221/222, the miR-200 family, and miR-125, which are widely detected in the heart and blood vessels, are deeply involved in the onset and progression of hypertensive cardiac injury by affecting endothelial cell proliferation, angiogenesis, cardiac dysfunction, endothelial inflammation, and other processes [11]. Notably, exercise training has been shown to promote angiogenesis and restore normal endothelial function and myocardial vascular function by regulating the expression level of miRNAs, thus effectively preventing and controlling hypertension and its complications [12]. However, studies assessing blood flow-restricted resistance training via cardiac transcriptome sequencing in rats with early hypertensive cardiac injury are lacking. Exploration in this area will provide important insights into the specific mechanisms by which blood flow-restricted resistance training ameliorates early hypertensive cardiac injury.

Therefore, the aim of this study was to compare the left ventricular myocardium of SHRs after blood flow-restricted resistance training intervention with that of SHRs in a quiet state by sequencing via transcriptomics sequencing technology, analyze the miRNAs that are differentially expressed in the hearts of the two groups of rats, analyze biological information, screen out the key miRNAs involved in blood flow-restricted resistance training to protect the early hypertensive heart, and further elucidate the effect of blood flow-restricted resistance training on the expression of these key miRNAs, with the goal of providing new ideas and strategies for the prevention and treatment of early hypertensive cardiac injury.

## 2. Materials and methods

### 2.1. Experimental animals and groups

All experimental rats, including four-week-old 48 SPF-grade male spontaneously hypertensive rats (SHRs) and 12 SPF-grade male Wistar Kyoto rats, weighing approximately 200 g, were purchased from Nanjing Qinglongshan Animal Breeding Base. The rats were housed in separate cages at an ambient temperature of 22 ± 2°C and a relative humidity of 30%−45% with a 12-hour light‒dark cycle (light hours: 08:30 a.m.-20:00 p.m.) and were fed ad libitum with water and food. The rats were randomly grouped into five groups of 12 rats each after one week of adaptive feeding: the normal group (WKY), hypertensive control group (SHR-SED), high-intensity resistance training group (HIRT), medium-intensity resistance training group (MIRT), and blood flow-restricted medium-intensity resistance training group (BFRT). All animal experimental operations were approved by the Ethics Committee for Animal Experiments of Nanjing Sports Institute (NO: DW-2023-01).

### 2.2. Exercise programme

Ladder climbing procedure: The exercise groups were trained on a ladder frame suitable for rat specifications, which had 54 vertical steps spaced 0.5 cm apart each, with a resting platform at the top of the steps to create a smooth environment for the animals to rest while climbing. All the animals were habituated to climbing behavior for 5 consecutive days prior to the maximum load test.

The maximum load (one rep max, 1RM) test consisted of an initial load of 75% of the body weight, attaching the load to the base of the rat’s tail, and after completing the first ladder climb, there was a 2-minute rest period before the next climb. During the next climb, the load was increased by another 15% of the body weight. This incremental process was repeated until the animal could not handle the load to complete the climb. To determine the maximum load, it was assumed that the animal should complete up to 6 climbs to avoid fatigue due to too many climbs.

Load selection and adjustment: After the maximal load test, resistance exercise training was carried out using the standardized value of the individual maximal load (load/body weight for the last complete climb) for each rat and adjusted weekly according to the animal’s body weight by adopting incremental resistance training with a gradual increase in load.

The training schedule was as follows: the exercise groups underwent 1–8 weeks of stair-climbing training, 5 days per week, 5 sets of stair-climbing were trained every day from 3–5 pm, 15 times for each set, the rats were stimulated to complete a single stair-climbing quickly by stimulating the tail tip, with a 1 min rest between each set, and the body weights and the maximal loads were measured at a fixed time every week.

Percentages of resistance load and blood flow restricted by blood flow-restricted medium-intensity resistance training: 5 days of training per week, weeks 1--2: 35% of the maximum load; weeks 3--5: 45% of the maximum load; weeks 6--8: 55% of the maximum load. At the same time as ladder climbing, rubber bands were used to loop the thigh root of the right lower limb of the rats, vascular blood flow-restricted resistance training was given, blood flow reperfusion was performed by lifting the blood flow restricted during a 1-min interval, and the rubber band looped the site again after rest. To better monitor the degree of blood flow restriction, small animal high-frequency color ultrasound (VisualSonics Inc., Canada) was used to monitor the blood flow velocity before and after ring ligature to achieve an optimal 30%−40% percentage of blood flow restriction as required for blood flow-restricted resistance training.

The resistance load for medium-intensity resistance training was as follows: 5 days per week, weeks 1--2: 35% maximal load; weeks 3--5: 45% maximal load; and weeks 6--8: 55% maximal load.

The resistance load for high-intensity resistance training was as follows: 5 days of training per week, 1–2:50% maximal load; 3–5:65% maximal load; and 6–8:75% maximal load.

The specific experimental protocol was described in previous studies [8].

### 2.3. Hemodynamic and cardiac function tests

A BP-2000 pressure tester (Beijing Softdragon Biological Co., Ltd., China) was used. Rat cardiac ultrasound: After 2%−3% isoflurane anesthesia, small animal high-frequency color ultrasound (Visual Sonics Inc., Canada) was used to assess the cardiac function of the rats, including ejection fraction (EF), fractional shortening (FS), MV E/A peak ratio (MV E/A), interventricular septal thickness at end-diastole (IVSd), interventricular septal thickness at end-systole (IVSs), left ventricular posterior wall end-diastolic thickness (LVPWd), left ventricular posterior wall end-systolic thickness (LVPWs), left ventricular internal diameter at end-diastole (LVIDd), left ventricular internal diameter at end-systole (LVIDs), and left ventricular mass (LV mass) [8].

### 2.4. Sample collection

The rats were fasted (without water) for 24 h at the end of 8 weeks of training, after which they were anesthetized with an intraperitoneal injection of 3% sodium pentobarbital (1 ml/kg) and dissected after anesthesia (disappearance of corneal reflexes and pain reflexes). Myocardial left ventricular tissue was removed and stored in a −80°C medical refrigerator for freezing for subsequent molecular biology examination.

### 2.5. RNAiso for extraction of total RNA

Myocardial left ventricular tissue stored at −80°C was transferred to a liquid nitrogen precooled mortar, continuously supplemented with liquid nitrogen and ground until powdered. A 100 mg/ml RNAiso for small RNA mixture was added to completely cover the sample, which was melted at room temperature and ground until the lysate was clear. The homogenate was allowed to stand for 5 min at room temperature and then centrifuged at 4°C and 12000 rpm for 5 min to collect the supernatant. The supernatant was collected by adding chloroform at a volume ratio of 1:5, emulsified by vigorous shaking for 15 s, and then centrifuged under the same conditions for 15 min after standing for 5 min. The colorless supernatant was mixed with an equal volume of isopropanol and then centrifuged for 10 min after standing at room temperature to obtain the RNA precipitate. The precipitate was washed with 1 ml of 75% ethanol and dried at room temperature for 5 min after centrifugation, the liquid was discarded, and the mixture was finally dissolved in RNase-free water and stored at −80°C [8].

### 2.6. MiRNA sequencing

The NEBNext Small RNA Library Prep Set for Illumina Kit (Cat. No. NEB#E7330S, NEB, USA) was used to construct miRNA libraries: 1 µg of total RNA was amplified via junction ligation, reverse transcription and PCR, the 140--160 bp product was purified via agarose gel electrophoresis, and 150 bp bipartite sequencing was completed via the Illumina NovaSeq 6000 platform after quality control (QC) on an Agilent 2100 Bioanalyzer. After the raw data were converted to raw reads via base recognition, reads containing 5’ primers, poly(A) tails, no 3’ junctions or tag sequences were filtered, and 15–41-nt high-quality clean reads were retained. For data analysis, known miRNAs were annotated, and redundant sequences were filtered via Bowtie software and sequentially compared to the Rfam v10.1, cDNA, Repbase and miRBase databases. Unannotated reads were subsequently predicted via miRDeep2 for novel miRNAs on the basis of the pre-miRNA hairpin structure and miRBase features. The differential expression miRNA screening criteria were p < 0.05 and |log2(fold change) | > 1, with/without biological duplicate samples, according to the R package DEG algorithm and Audic Claverie algorithm, respectively. Target gene prediction was performed via miranda software (S ≥ 150, ΔG ≤ −30 kcal/mol, demand strict 5’ seed pairing), and target gene functions were analyzed via the R package hypergeometric distribution method for GO and KEGG enrichment [8].

### 2.7. RNA extraction and qRT‒PCR

#### 2.7.1. RNA extraction and concentration determination.

RNA was extracted as described above. The concentration was determined after RNA extraction by adding 2 µl of TE buffer solution dropwise to the spectrophotometer and zeroing. Then, 2 µl of RNA stock solution was added dropwise to the spectrophotometer microtiter plate, and its absorbance and concentration at 260 nm and 280 nm of the spectrophotometer were read to determine the concentration and purity of the RNA mixture.

#### 2.7.2. Reverse transcription.

Reverse transcription was performed via the PrimeScript™ RT Master Mix (Perfect Real Time) (RR036A) kit: 1 µg of RNA template was reconstituted on ice and centrifuged to collect the residue, and the reverse transcription reaction system was prepared according to the proportions described in the instructions (S1 Table) and then mixed and centrifuged to run the amplification program in a PCR instrument (S2 Table). After cDNA synthesis, 2 µl of cDNA was diluted with 98 µl of RNase-free dH2O to a final concentration of 2 ng/µl for qRT‒PCR, and the rest of the cDNA was stored at −20°C.

#### 2.7.3. miRNA qRT‒PCR.

The sequences of primers used for qRT‒PCR with the miRNA kit are shown below (S3 Table).

Amplification was performed via the Mir-X miRNA qRT‒PCR TB Green® Kit (638314): the primers for the target genes were diluted to 10 µM in RNase-free dH2O, and a reaction system containing 2 ng/µl cDNA working solution was prepared according to the instructions (S4 Table), mixed, centrifuged, and then dispensed into a 96-well plate (25 µl/well, triple replicate wells). After the plate was sealed, the mixture was centrifuged at 1000 rcf for 2 min to remove air bubbles, and the reaction was carried out on a Step One@ Real-Time PCR System (S5 Table). At the end of the reaction, the exported data were used to quantify the qRT‒PCR results via ^△△^Ct values, and the relative quantitative analysis was performed via the 2^-△△^Ct method, ^△△^Ct = ^△^Ct experimental group - ^△^Ct control group. The differences in the expression of target genes were expressed as 2^-△△^Ct values, the expression of the target gene miRNAs was calculated in relation to that of the internal reference gene U6, and the changes in the expression of target gene miRNAs in the various groups were determined [9].

### 2.8. Data analysis

The qRT‒PCR data were used to calculate the relative miRNA content of the samples via the 2^-△△^Ct method, and GraphPad Prism 8.0 Demo software was used for graphing. All the data were statistically analyzed via SPSS 22.0 and are expressed as the means ± standard errors (means ± SEMss). Weight, blood pressure and cardiac function data were analyzed by two-way repeated-measures ANOVA. Bonferroni multiple comparisons were performed after ANOVA to compare the differences in body weight, blood pressure and cardiac function data between the groups in the pre- and postexperimental time periods. One-way ANOVA between groups was performed on qRT‒PCR data that conformed to a normal distribution, and Levene’s test was performed to test the chi-square test of variance and to compare the differences in each data point between groups. P < 0.05 was considered a significant difference.

## 3. Results

### 3.1. Changes in the body weights of the rats before and after training

The results of body weight measurements revealed that there was no significant difference in the comparison of rat body weight between the groups before training (P > 0.05); after training, the body weight of the rats in all the groups increased significantly (P < 0.05) compared with that before training, with the most significant increase in body weight in the HIRT group (P < 0.05), and the change in body weight gain between the other groups was not significantly different (P > 0.05) (Table 1).

**Table 1:** Changes in SHR body weight, hemodynamics and cardiac function before and after training.

	WKY	SHR-SED	HIRT	MIRT	BFRT
**Initial**					
** BW (g)**	201.13 ± 0.60	199.93 ± 0.90	198.29 ± 0.91	200.98 ± 0.69	201.14 ± 0.46
** HR**	326.27 ± 18.89	394.77 ± 50.85^e^	407.33 ± 5.85^e^	412.23 ± 18.15^e^	411.11 ± 18.12^e^
** SBP (mmHg)**	151.43 ± 11.14	171.78 ± 20.82^e^	171.72 ± 20.59^e^	178.32 ± 13.35^e^	177.04 ± 14.83^e^
** DBP (mmHg)**	71.71 ± 14.13	82.75 ± 15.70^e^	95.02 ± 22.01^e^	94.61 ± 19.55^e^	91.17 ± 19.24^e^
** EF (%)**	71.18 ± 3.77	60.64 ± 5.26^e^	60.52 ± 4.43^e^	60.31 ± 6.58^e^	58.54 ± 5.15^e^
** FS (%)**	41.57 ± 3.11	33.58 ± 3.78^e^	33.42 ± 3.28^e^	33.50 ± 5.04^e^	32.29 ± 3.78^e^
** MV E/A**	1.60 ± 0.11	1.48 ± 0.06^e^	1.49 ± 0.03^e^	1.47 ± 0.05^e^	1.49 ± 0.05^e^
** IVSd (mm)**	1.49 ± 0.08	1.52 ± 0.06	1.51 ± 0.10	1.49 ± 0.06	1.51 ± 0.03
** IVSs (mm)**	2.42 ± 0.04	2.41 ± 0.04	2.41 ± 0.03	2.43 ± 0.03	2.42 ± 0.02
** LVIDd (mm)**	6.65 ± 0.40	7.32 ± 0.57^e^	7.44 ± 0.57^e^	7.51 ± 0.64^e^	7.45 ± 0.47^e^
** LVIDs (mm)**	3.90 ± 0.43	4.86 ± 0.49^e^	4.96 ± 0.52^e^	5.00 ± 0.67^e^	5.04 ± 0.36^e^
** LVPWd (mm)**	1.57 ± 0.06	1.57 ± 0.07	1.57 ± 0.06	1.58 ± 0.11	1.60 ± 0.09
** LVPWs (mm)**	2.51 ± 0.04	2.74 ± 0.10^e^	2.76 ± 0.25^e^	2.78 ± 0.22^e^	2.75 ± 0.06^e^
** LV Mass (mg)**	556.06 ± 35.34	636.30 ± 48.28^e^	627.99 ± 47.12^e^	632.80 ± 21.27^e^	639.69 ± 63.37^e^
**Final**					
** BW (g)**	275.98 ± 16.24^a^	267.67 ± 17.64^a^	288.49 ± 16.29^abe^	269.11 ± 17.99^ac^	271.55 ± 22.95^ac^
** HR**	317.92 ± 25.98	402.27 ± 46.27^e^	395.51 ± 9.24^e^	361.86 ± 48.13^abce^	341.23 ± 36.11^abc^
** SBP (mmHg)**	146.22 ± 9.81	179.20 ± 13.04^e^	196.97 ± 10.46^abe^	154.41 ± 18.90^abc^	149.16 ± 22.91^abc^
** DBP (mmHg)**	64.31 ± 8.05	98.76 ± 7.69^ae^	130.54 ± 20.99^abe^	80.89 ± 14.93^abce^	64.21 ± 8.28^abcd^
** EF (%)**	71.15 ± 4.17	59.13 ± 3.05^e^	68.12 ± 3.03^ab^	67.97 ± 5.15^ab^	68.39 ± 1.86^ab^
** FS (%)**	41.64 ± 3.58	32.49 ± 2.14^e^	39.15 ± 2.43^ab^	39.03 ± 4.23^ab^	39.20 ± 1.47^ab^
** MV E/A**	1.59 ± 0.05	1.46 ± 0.03^e^	0.57 ± 0.04^ab^	1.61 ± 0.02^abc^	1.62 ± 0.03^abc^
** IVSd (mm)**	1.49 ± 0.08	1.61 ± 0.02^ae^	1.51 ± 0.06^b^	1.51 ± 0.06^b^	1.52 ± 0.03^b^
** IVSs (mm)**	2.45 ± 0.03	2.54 ± 0.03^ae^	2.44 ± 0.04^b^	2.44 ± 0.08^b^	2.43 ± 0.10^b^
** LVIDd (mm)**	6.80 ± 0.51	7.50 ± 0.42^e^	6.79 ± 0.24^ab^	6.75 ± 0.12^ab^	6.69 ± 0.47^ab^
** LVIDs (mm)**	3.98 ± 0.45	5.07 ± 0.34^e^	4.13 ± 0.22^ab^	4.12 ± 0.30^ab^	4.07 ± 0.31^ab^
** LVPWd (mm)**	1.55 ± 0.11	1.66 ± 0.06^ae^	1.71 ± 0.07^ae^	1.56 ± 0.09^bc^	1.57 ± 0.09^bc^
** LVPWs (mm)**	2.47 ± 0.17	2.73 ± 0.14^e^	2.72 ± 0.25^e^	2.57 ± 0.12^abc^	2.55 ± 0.09^abc^
** LV Mass (mg)**	549.20 ± 31.59	693.43 ± 64.45^ae^	679.23 ± 49.74^ae^	540.93 ± 40.45^abc^	556.50 ± 46.45^abc^

The values are mean ± SEM. n = 12 rats in each group. BW: Body weight; HR: Heart rate; SBP: Systolic blood pressure; DBP: Diastolic blood pressure; EF: Ejection fraction; FS: Fraction shortening; MV E/A: E peak/ A peak of mitral valve; IVSd: Interventricular septal thickness at end–diastole; IVSs: Interventricular septal thickness at end–systole; LVIDd: Left ventricular internal diameter at end–diastole; LVIDs: Left ventricular internal diameter at end–systole; LVPWd: Left ventricular posterior wall end–diastolic thickness; LVPWs: Left ventricular posterior wall end– systolic thickness; LV Mass: Left ventricular mass. ^a^P < 0.05 vs Before training; ^b^P < 0.05 vs SHR-SED; ^c^P < 0.05 vs HIRT; ^d^P < 0.05 vs MIRT; ^e^P < 0.05 vs WKY.

### 3.2. Effects of different types of resistance training on hemodynamics and cardiac function in SHRs

Compared with the SHR-SED group, the BFRT group and the MIRT group effectively lowered blood pressure of SHRs (P < 0.05), while the HIRT group significantly increased blood pressure of SHRs, and the increase in DBP in the HIRT group was more significant (P < 0.05). Compared with the WKY group, the MIRT group significantly increased blood pressure of SHRs (P < 0.05), while there was no significant difference in blood pressure of SHRs in the BFRT group (P > 0.05) (Table 1).

Compared with the SHR-SED group, the BFRT and MIRT groups were able to improve the EF, FS, and MV E/A of SHRs; reduce the HR; and decrease the IVSd, IVSs, LVIDd, LIVDs, LVPWd, LVPWs, and LVMass (P < 0.05), and there was no significant difference between the two groups’ improvement effects (P > 0.05). Compared with the SHR-SED group, the HIRT group did not reduce the HR or reduce the LVPWs and LV mass (P > 0.05), although it improved the EF, FS, and MV E/A in SHRs and decreased the IVSd, IVSs, LVIDd, and LIVDs (P < 0.05). The BFRT group and the MIRT group were more effective at improving LV compliance, reducing the HR, and attenuating the LVPW and LV mass better than the HIRT group was (P < 0.05). Additionally, compared with the WKY group, there were no significant differences in cardiac function indicators between the BFRT group and the MIRT group, and there were no significant differences between the two groups (P > 0.05) (Table 1).

### 3.3. Results of sequencing data quality assessment

The raw reads were filtered, the sequencing error rate was checked, and the GC content distribution was checked to obtain clean reads for subsequent analysis. The results of the sequencing data quality assessment revealed that the miRNA sequencing error was 0.01%, the Q20 of all the samples was greater than 96%, the Q30 was greater than 94%, and the GC content was close to the theoretical value of 50%, which indicated that the data quality was high and could satisfy the requirements of the subsequent analysis in this study, as shown in Table 2.

**Table 2:** Summary of miRNAs sequencing data quality.

Sample Name	Raw reads	Clean reads	Bases (G)	Error rate (%)	Q20 (%)	Q30 (%)	GC (%)
**SHR1**	21738765	21128151	1.94	0.01	97.24	95.43	48.32
**SHR2**	21906472	21442185	1.85	0.01	97.90	95.37	49.90
**SHR3**	21377510	21677363	1.79	0.01	96.92	95.15	47.22
**BFRT1**	21413123	21009676	1.92	0.01	98.18	96.23	49.48
**BFRT2**	21692869	20974460	1.78	0.01	96.73	94.40	48.39
**BFRT3**	21603232	21151377	1.86	0.01	97.96	95.57	48.59

Q20: Percentage of bases with Phred value greater than 20; Q30: Percentage of bases with Phred value greater than 30; GC: Percentage of bases G and C in the filtered sequence fragments. (The quality of sequencing data is usually judged as follows: Q20 > 90%, Q30 > 85% for a satisfactory sequencing error rate, and GC in the range of 45%−55%.)

### 3.4. Results of comparison with the reference genome

After quality assessment of the sequencing data of each sample and comparison with the reference genome, the total comparison rate of each sample of miRNA was greater than 94%, and the credibility of the data was good and suitable for subsequent analyses (Table 3).

**Table 3:** Table of miRNA comparisons with reference genomes.

Sample Name	Total reads	Aligned reads	Aligned (%)
**SHR1**	21128151	20356531	96.35
**SHR2**	21442185	20268331	94.53
**SHR3**	21677363	20820927	96.05
**BFRT1**	21009676	20037340	95.37
**BFRT2**	20974460	20088553	95.78
**BFRT3**	21151377	20383983	96.37

When Aligned is greater than or equal to 85%, it indicates that the credibility of the sequencing results is high.

### 3.5. Results of miRNA differential expression analysis

**Fig 1:**
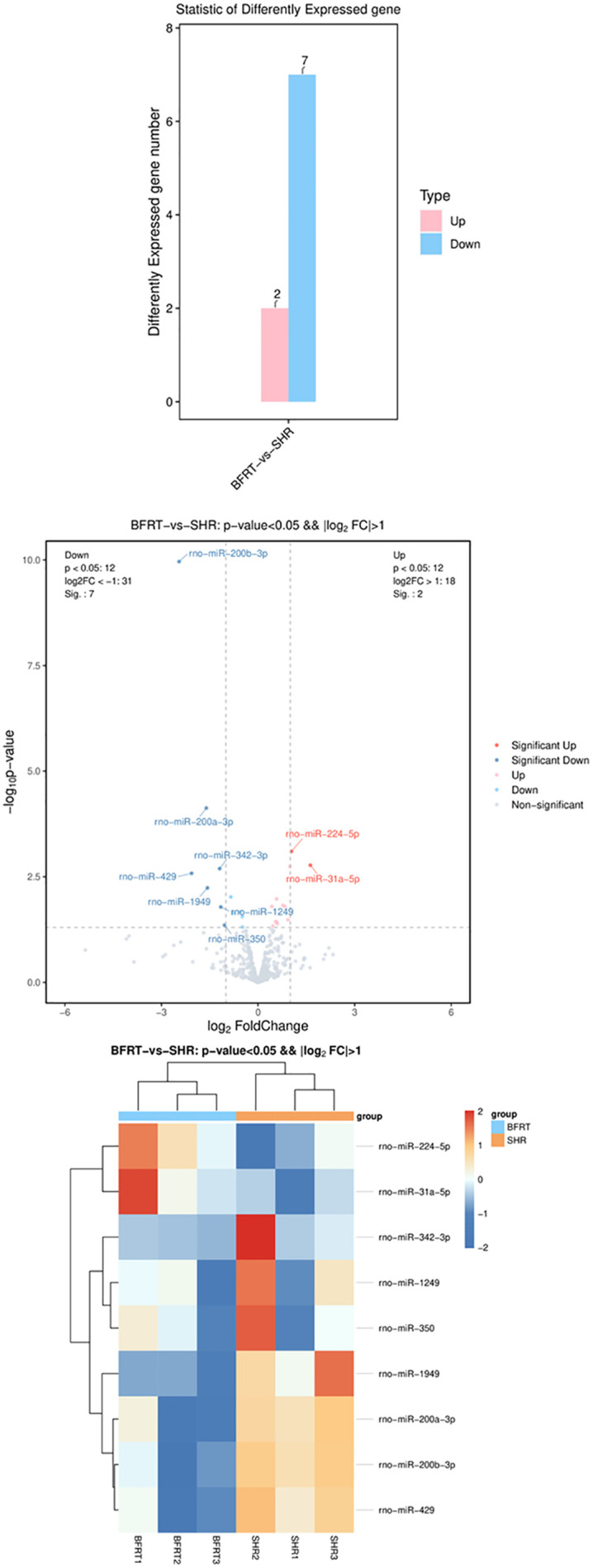
Volcano plot and clustering of differentially expressed miRNAs. Histograms, volcano plots and cluster plots were all generated using the Oebiotech Cloud platform (cloud.oebiotech.cn).

The results of miRNA differential expression analysis revealed that, with P < 0.05 and |log2FoldChange| ≥ 1 as the criterion for differential significance, nine differentially expressed miRNAs were present in the SHR-SED group compared with those in the BFRT group, of which the expression of two miRNAs (miR-224-5p and miR-31a-5p) was significantly upregulated, and the expression of seven miRNAs (miR-200b-3p, miR-200a-3p, miR-342-3p, miR-429, miR-1949, miR-1249, and miR-350) was significantly downregulated. Among these nine genes, the most prominently differentially expressed gene was miR-200b-3p (Table 4, Fig 1).

**Table 4:** Differential expression of miRNAs in SHR-SED group and BFRT group (all miRNAs).

miRNA	Expression BFRT	Expression SHR	log2 Fold Change	p-value	Regulation
**miR-224-5p**	236.89	100.03	1.04	0.00	Up
**miR-31a-5p**	6.12	1.68	1.63	0.00	Up
**miR-452-5p**	22.56	9.75	0.99	0.00	Up
**miR-142-3p**	40.51	23.33	0.58	0.01	Up
**miR-598-3p**	4.83	2.47	0.77	0.02	Up
**miR-21-5p**	3627.93	2329.11	0.43	0.02	Up
**miR-142-5p**	5.49	2.62	0.84	0.02	Up
**miR-434-3p**	5.37	2.40	0.93	0.03	Up
**miR-199a-5p**	667.88	393.90	0.55	0.04	Up
**miR-374-3p**	9.62	5.56	0.58	0.04	Up
**miR-338-3p**	7.45	4.23	0.60	0.04	Up
**miR-148a-3p**	52.14	32.52	0.46	0.05	Up
**miR-200b-3p**	3.25	15.37	−2.45	0.00	Down
**miR-200a-3p**	2.09	5.47	−1.61	0.00	Down
**miR-342-3p**	7.48	14.65	−1.19	0.00	Down
**miR-429**	0.33	1.20	−2.06	0.00	Down
**miR-1949**	0.54	1.41	−1.57	0.01	Down
**miR-184**	4.84	7.72	−0.84	0.01	Down
**miR-1249**	2.54	4.80	−1.16	0.02	Down
**miR-182**	14.05	17.75	−0.55	0.02	Down
**miR-664-3p**	4.71	6.85	−0.76	0.02	Down
**miR-22-5p**	122.24	150.32	−0.49	0.03	Down
**miR-350**	2.47	4.29	−1.04	0.04	Down
**miR-92a-3p**	9.24	11.23	−0.48	0.05	Down

### 3.6. GO functional enrichment analysis of differential miRNA target genes

**Fig 2:**
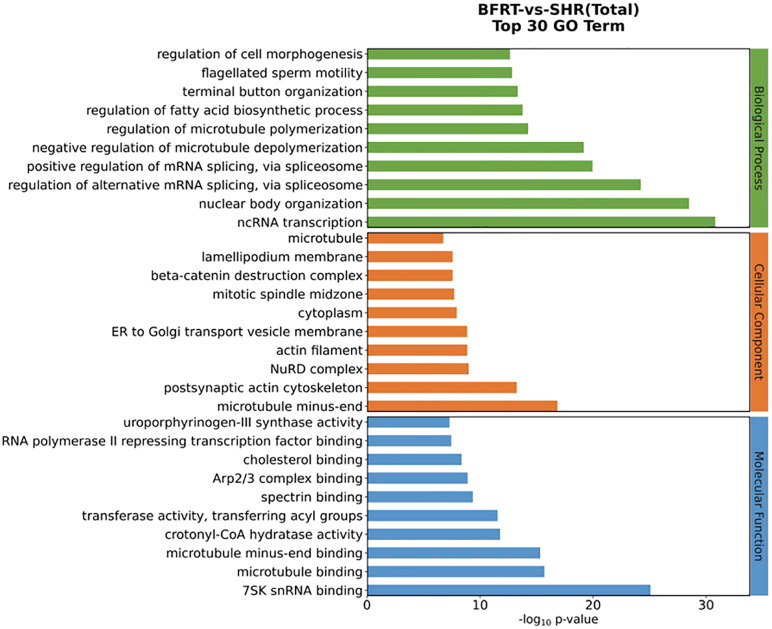
Histogram of the GO functional enrichment of differential miRNA target genes. Histogram was generated using the Oebiotech Cloud platform.

The results of the GO functional enrichment analysis of the differential miRNA target genes included biological process (BP), cellular component (CC) and molecular function (MF) terms (Fig 2, S6 Table). The top 10 GO terms (P < 0.05) under the three major classifications after GO functional enrichment are shown in Fig 2. Among them, the top 5 with the highest BP enrichment were ncRNA transcription, nuclear body organization, alternative mRNA splicing regulation via the spliceosome, positive regulation of mRNA splicing via the spliceosome, and microtubule depolymerization; the top 5 with the highest CC enrichment were microtubule minus-end, postsynaptic actin cytoskeleton, NuRD complex, actin filament, and ER to Golgi transport vesicle membrane; and the top 5 with the highest MF enrichment were 7SK snRNA binding, microtubule binding, microtubule minus-end binding, crotonyl-CoA hydratase activity, transferase activity, and acyl group transfer.

### 3.7. Differential miRNA target gene KEGG/Reactome/WikiPathways pathway analysis

**Fig 3:**
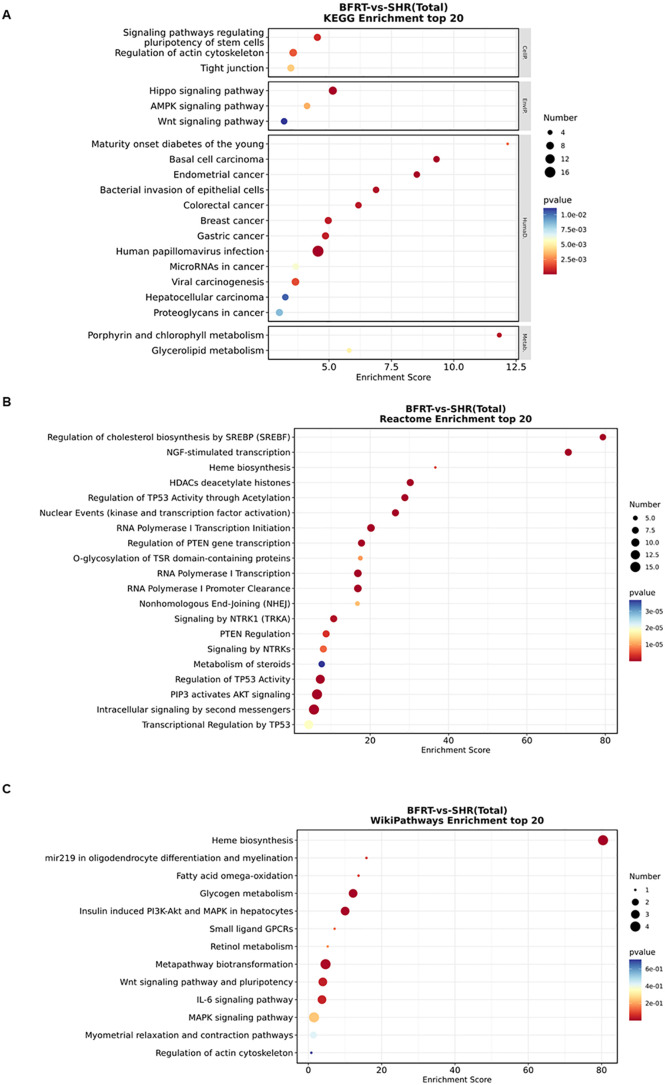
Differential miRNA target gene pathway enrichment bubble map. (A) Differential miRNA target gene KEGG pathway enrichment bubble map; (B) differential miRNA target gene Reactome pathway enrichment bubble map; (C) differential miRNA target gene WikiPathways pathway enrichment bubble map. Bubble maps were generated using the Oebiotech Cloud platform.

The results of the differential miRNA target gene KEGG/ Reactome/ WikiPathways pathway analysis are shown in Fig 3, which shows the top 20 KEGG/Reactome/WikiPathways pathways that were relevant and significantly enriched for this study: the Hippo signaling pathway, signaling pathways regulating pluripotency of stem cells, the AMPK signaling pathway, the Wnt signaling pathway, etc.; the PIP3 pathway activated AKT signaling, signaling by NTRK1, PTEN regulation, heme biosynthesis, and signaling by NTRKs; and the WikiPathways included heme biosynthesis, insulin-induced PI3K-Akt and MAPK in hepatocytes, the Wnt signaling pathway and pluripotency, the IL-6 signaling pathway, the MAPK signaling pathway, etc. (P < 0.05). In the bubble map, the higher the enrichment score, the greater the degree of enrichment; P < 0.05 for significant enrichment, the darker the color, the greater the significance of the enrichment; the number of genes enriched in the pathway entry, the larger the radius of the circle, the greater the number of genes enriched.

### 3.8. qRT-PCR results of differentially expressed miRNAs in rats’ myocardial tissue after training

**Fig 4:**
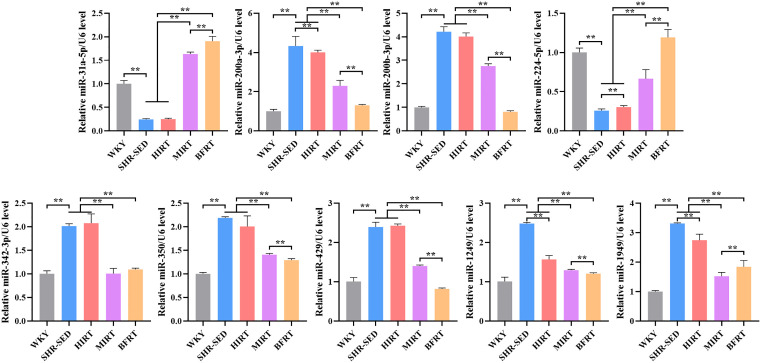
qRT-PCR results of differentially expressed miRNAs in rats’ myocardial tissue after training. Histograms were generated using GraphPad Prism 8.0 Demo software. The values are mean ± SEM. n = 8 rats in each group. **P < 0.01.

Compared with the SHR-SED group, the BFRT group and the MIRT group significantly reduced the expression levels of miR-200a-3p, miR-200b-3p, miR-342-3p, miR-350, miR-429, miR-1249, miR-1949 in SHR myocardium, and increased the expression levels of miR-31a-5p and miR-224-5p (P < 0.01). Among these, except for miR-342-3p, the changes in miRNA expression levels in the BFRT group were more significant (P < 0.01). The HIRT group significantly reduced the expression levels of miR-200a-3p, miR-1249, and miR-1949 in SHR myocardium, and increased the expression level of miR-224-5p (P < 0.01) (Fig 4). The validation results from qRT-PCR were consistent with the sequencing results, indicating that BFRT effectively regulated the expression levels of miR-200a-3p, miR-200b-3p, miR-342-3p, miR-350, miR-429, miR-1249, miR-1949, miR-31a-5p, and miR-224-5p in SHR myocardium.

## 4. Discussion

This study found that in simple resistance training, high-intensity resistance training increased SHR blood pressure, while medium-intensity resistance training lowered blood pressure and heart rate. However, compared with normal rats, the blood pressure and heart rate of SHRs in the medium-intensity resistance training group remained high. The BFRT group not only lowered SHR blood pressure and heart rate, but also showed no significant difference compared with normal rats. Compared with medium-intensity resistance training, BFRT showed a more significant decrease in blood pressure and heart rate. In terms of cardiac function, the BFRT group and the MIRT group were able to improve both the function and structure of the SHR heart, while the HIRT group seemed to focus on improving SHR cardiac function, with the thickness and mass of the left ventricular posterior wall still showing signs of hypertrophy. At the same time, previous work in this study also revealed that blood flow-restricted resistance training provides more benefits than simple resistance training in the protection of early hypertensive cardiac structure and function [8]. Therefore, in this study, transcriptomic sequencing and standardized analysis were performed on left ventricular myocardial tissue from the SHR-SED group and the BFRT group of SHRs. The results revealed nine differentially expressed miRNAs (two miRNAs were significantly upregulated, and seven miRNAs were significantly downregulated). Among these, the miRNAs upregulated after BFRT were miR-224-5p and miR-31a-5p, while the downregulated miRNAs were miR-200b-3p, miR-200a-3p, miR-342-3p, miR-429, miR-1949, miR-1249, and miR-350.

### 4.1. Effects of blood flow-restricted resistance training on miRNAs from the left ventricular myocardium in spontaneously hypertensive rats

Among the 9 differentially expressed miRNAs, miR-224-5p can target and regulate endothelial cell proliferation, migration, and inflammatory responses and is also associated with tumor development and heart failure. Study has shown that miR-224-5p can be activated by SOX17, inhibiting the expression and release of downstream NR4A3 and PCSK9, thereby improving endothelial function [13]. Additionally, in pulmonary fibrosis tissue and vascular endothelial tissue from a pulmonary arterial hypertension model, miR-224-5p expression is downregulated, but its expression levels increase after treatment with HDAC inhibitors [14]. Whereas in plasma from a heart failure model, miR-224-5p expression is upregulated, and it is believed to regulate iron metabolism and oxidative stress dysregulation-induced heart failure through the circSnx12/miR-224-5p/FTH1 pathway [15]. In this study, miR-224-5p expression was downregulated in the SHR myocardium, which is consistent with the findings in the pulmonary arterial hypertension model. However, owing to differences in the tissue samples used for sequencing, we speculate that miR-224-5p expression is associated not only with pathological models but also with cardiac injury and the progression of pathological development.

miR-31a-5p has been confirmed to be a negative regulator of epithelial-to-mesenchymal transition in cardiac fibrosis, and miR-31a-5p also plays a complex role in cell proliferation. In the hearts of newborn rats, miR-31a-5p targets RhoBTB1, activates the expression of PCNA, EdU, and Ki-67, and promotes myocardial cell proliferation and cardiac regeneration [16]. Following myocardial infarction-induced heart failure in rats, miR-31a-5p expression is downregulated, leading to Ca²⁺ decay. However, after endurance training, miR-31a-5p expression is upregulated in rat myocardial tissue, prolonging Ca²⁺ decay and improving cardiac function [17]. In this study, miR-31a-5p expression was downregulated in the SHR myocardium, which is consistent with the findings of previous studies. These findings suggest that miR-31a-5p may directly influence myocardial cell function regardless of the physiological environment.

miR-342-3p regulates cell apoptosis and autophagy. A study has shown that miR-342-3p can target and downregulate the expression of SOX6 and TFEB, thereby inhibiting myocardial cell apoptosis and autophagy, and exerting a protective effect on myocardial cells in mice with heart failure induced by myocardial infarction [18]. Additionally, other study has reported that levels of miR-342-3p are reduced in the plasma of heart failure mice, but after treatment with an AT1 receptor blocker, miR-342-3p expression levels recover, and the prognosis of heart failure in mice improves [19]. Furthermore, miR-342-3p is believed to potentially act as a cardioprotective factor, with its mechanism of action associated with SGLT2 inhibition [20]. However, in this study, the expression of miR-342-3p was upregulated in the myocardia of SHRs, which contradicts previous findings. Therefore, further research is needed to validate the expression of miR-342-3p in different cardiac injury models.

miR-200b-3p, miR-200a-3p, and miR-429 belong to the miR-200 family, are expressed primarily in endothelial cells, and are associated with proliferation and endothelial‒mesenchymal transition processes [21]. A study has reported that in the placenta and plasma of hypertensive patients, the expression levels of miR-200 family members are increased, while ZEB1 expression is decreased. It is proposed that miR-200 family members promote vascular endothelial damage by regulating ZEB1/ZEB2 expression, thereby triggering hypertensive symptoms [22]. Furthermore, in the aorta of atherosclerotic mice and in ox-LDL-treated HAECs, increased miR-429 expression has been shown to target and inhibit Bcl-2, leading to enhanced endothelial cell apoptosis and vascular injury [23]. In the myocardium and pulmonary aorta of rats with pulmonary arterial hypertension, the expression of miR-200a-3p is upregulated, inhibiting the expression of IGF1R and PDCD4. After melatonin treatment, the H19-miR-200a-PDCD4 signalling pathway is activated, inhibiting cell proliferation and alleviating PAH symptoms [24]. Moreover, members of the miR-200 family can participate in cell proliferation, differentiation, and apoptosis. In type 2 diabetic mouse models, miR-200b and miR-429 mimics can target and downregulate ZEB1, upregulate COX-2 and MCP-1, increase the expression of inflammatory factors in myocardial tissue, promote myocardial cell apoptosis, and cause myocardial damage, leading to cardiac disease [25]. In HUVECs, overexpression of miR-200b-3p reduces HDAC4 and VEGF expression, promotes oxidative stress-induced endothelial cell damage, leading to increased HUVEC apoptosis [26]. Additionally, some evidence suggests that the miR-200 family participates in ROS production, contributing to redox imbalance and the development of hypertension characterized by oxidative stress-dependent endothelial dysfunction [27]. The sequencing results obtained in this study are consistent with those of previous studies, which revealed that miR-200b-3p, miR-200a-3p, and miR-429 are upregulated in the myocardia of SHRs. However, other studies have reported that in myocardial fibrosis patients, miR-200 family members are among the top differentially downregulated transcripts according to the results of transcriptomic sequencing of myocardial tissue [28]. In rats with myocardial injury caused by myocardial infarction, the expression of miR-200b-3p and miR-200a-3p is downregulated, and melatonin can regulate the miR-200b-3p/HMGB1 axis to alleviate inflammation and heart failure caused by myocardial infarction [29]. Therefore, we believe that the expression of miR-200 family members varies across different cardiac injury models and requires further validation. In myocardial injury, which is characterized primarily by endothelial dysfunction in a hypertension model, the expression of miR-200 family members is upregulated.

miR-1949 plays crucial roles not only in cell proliferation and differentiation but also in tumorigenesis and organ development. In the lung tissue of newborn rats, miR-1949 expression is downregulated [30], whereas in the bladder tissue of bladder cancer rats and the kidney tissue of kidney-injured rats, miR-1949 expression is upregulated, which is believed to be closely related to the severity of inflammatory damage [31]. However, there are no previous studies on the expression of miR-1949 in cardiac injury. In this study, the expression of miR-1949 in the SHR myocardium was upregulated, which is consistent with previous findings of miR-1949 expression in pathological states.

miR-1249 is a gene associated with autophagy that regulates the proliferation and apoptosis of endothelial cells. In the pulmonary arteries of rats with pulmonary arterial hypertension, miR-1249 expression is upregulated, promoting excessive proliferation of pulmonary arterial smooth muscle cells and endothelial cells by inhibiting the HDAC10/NFκB/CaSR cascade, maintaining their anti-apoptotic state, and accelerating the progression of pulmonary arterial hypertension. It is considered a key factor in causing endothelial damage and dysfunction [32]. In rats with left ventricular dysfunction caused by hypoxia, miR-1249 expression is also upregulated, leading to the induction and activation of LC3/Caspase3 expression, which is believed to activate autophagy and lead to myocardial cell apoptosis and impaired cardiac function [33]. In this study, miR-1249 expression was upregulated in the myocardia of SHRs, which is consistent with previous findings.

miR-350 is a highly relevant miRNA induced by stress overload-induced hypertrophy and serves as a key regulatory factor in pathological cardiac hypertrophy and apoptosis in rats. Study results indicate that in the rat myocardium with impaired diastolic function due to oxidative stress, miR-350 expression is upregulated, and following intervention therapy, miR-350 expression subsequently decreases. miR-350 enhances the antioxidant capacity of rat myocardium by regulating the activity of Nrf-2, CAT, and GSH-Px, thereby protecting the heart [34]. In the myocardium of rats with pathological hypertrophy, miR-350 expression is also upregulated, and it inhibits p38 and JNK protein synthesis through the MAPK pathway, inducing myocardial cell hypertrophy [35]. Additionally, miR-350 expression is also upregulated in myocardial tissue from a myocardial ischemia–reperfusion injury model. However, after chelating miR-350 with lncRNA AK020546, ErbB3, Bcl-2, and AKT expression is activated, Bad phosphorylation is inhibited, and myocardial cell apoptosis is reduced [36]. Although previous studies have not clearly established the expression of miR-350 in hypertensive model myocardial tissue, the upregulation of miR-350 in SHR myocardial tissue observed in this study is consistent with the conclusions of the aforementioned studies.

In summary, on the basis of previous studies and the miRNA sequencing data from this study, in the hearts of spontaneously hypertensive rats, miR-224-5p and miR-31a-5p may act as protective factors against myocardial injury, whereas miR-200b-3p, miR-200a-3p, miR-342-3p, miR-429, miR-1949, miR-1249, and miR-350 may act as myocardial injury-inducing factors. After 8 weeks of BFRT intervention improved cardiac function in SHRs, miR-224-5p and miR-31a-5p were upregulated, whereas miR-200b-3p, miR-200a-3p, miR-342-3p, miR-429, miR-1949, miR-1249, and miR-350 were downregulated.

### 4.2. Pathway analysis of differentially expressed miRNAs

The target genes of the differentially expressed miRNAs obtained from sequencing were subjected to GO functional enrichment and KEGG/Reactome/WikiPathways pathway analyses to predict their functions. The results revealed that the main functional enrichment was negative regulation of microtubule depolymerization, microtubule binding, etc. Pathway analysis focused primarily on the PI3K‒Akt signaling pathway, the MAPK signaling pathway, etc. A literature review revealed that these signaling pathways are indeed closely associated with the onset and progression of hypertension-induced cardiac dysfunction.

Among these pathways, the PI3K‒Akt signaling pathway plays a multifaceted regulatory role in biological processes, including the regulation of telomerase activity, control of cell apoptosis, modulation of cell invasiveness, dynamic management of the cell cycle, and promotion of angiogenesis. This pathway can influence and regulate multiple key downstream protein targets, such as mTOR, NF-κB, GSK-3β, eNOS, and members of the Bcl2 family, thereby achieving precise regulation of cellular physiological and pathological processes [37]. In hypertension research, after long-term aerobic exercise, Pi3k is effectively activated in SHR vessels and shows a significant upward trend, thereby promoting the phosphorylation of downstream Akt, activating related signaling pathways, and reducing SBP [38]. Similarly, moderate-intensity resistance exercise can also activate PI3K, upregulate p-Akt, activate the pathway, significantly reduce myocardial fibrosis, and improve cardiac remodeling function [39]. These effects are attributed to eNOS downstream of the PI3K‒Akt signaling pathway. Activation of the PI3K‒Akt pathway induces the upregulation of eNOS expression and stimulates eNOS phosphorylation, leading to NO release. eNOS-induced NO not only influences collagen deposition and cardiac fibroblast activity but also affects cardiac endothelial cell function, regulating cardiac vascular regeneration and fibrosis development [40]. In this context, studies have shown that in SHRs with myocardial ventricular hypertrophy, cellular hypertrophy, fibrosis, and myocardial cell apoptosis, the PI3K-Akt-eNOS pathway in the myocardium is inhibited. After treatment with telmisartan, myocardial remodeling in SHRs improved, and the expression of proteins in the PI3K-Akt-eNOS pathway increased [41]. Other studies have also indicated that fucoidan can improve myocardial vascular dysfunction in hypertensive rats through a PI3K-Akt-eNOS-dependent mechanism [42]. These studies demonstrate that the PI3K‒Akt pathway plays a crucial role in hypertensive cardiac injury.

MAPK is a cytoplasmic signal transduction protein. After being specifically phosphorylated, it is activated and can regulate various cellular processes, such as cell growth and cell size regulation. The MAPK pathway commonly includes the Ras/Raf/ERK pathway, the JNK pathway, and p38 MAPK.

In mouse and human models of pathological myocardial hypertrophy leading to cardiac dysfunction, ERK1/2, SAPK/JNK, and p38 MAPK are activated and increased in the heart, and these pathways further increase the expression of inflammatory mediators (TNF, IL-6) and hypertrophic factors (ET-1) [43]. In studies related to hypertension-induced cardiac injury, MAPK signaling pathways are upregulated in hypertensive mice with myocardial hypertrophy, fibrosis, and heart failure. Treatment with a MAPK inhibitor (Doramapimod) reduces blood pressure and improves cardiac function in hypertensive mice [44]. Other studies have shown that in mechanically stretched rat models of pulmonary arterial hypertension, the MAPK pathway is activated, mediating increased TGFβ-1 expression and leading to vascular remodeling and impaired cardiac function [45]. The use of p38 MAPK inhibitors reduces MAPK/TGFβ-1 expression in the myocardium of pressure-loaded mouse models, thereby inhibiting myocardial fibrosis and restoring cardiac function [46]. These findings confirm that the MAPK pathway plays a promotional role in myocardial fibrosis and hypertrophy in hypertensive cardiac injury and that inhibiting the activation of the MAPK pathway can serve as a therapeutic target for treating hypertensive cardiac injury.

### 4.3. Effects of blood flow-restricted resistance training on miR-200b-3p expression levels in the SHR myocardium

On the basis of the above results and discussion, we found that among the differentially expressed miRNAs, miR-200b-3p presented the most significant expression differences. miR-200b-3p is closely associated with endothelial cell function, inflammation, and myocardial cell proliferation and apoptosis in the heart [26]. Moreover, we detected that the expression of miR-200b-3p in the myocardium after blood flow-restricted resistance training improved early SHR cardiac function and that miR-200b-3p expression was downregulated and significantly different from that in the other exercise groups. In summary, we speculate that miR-200b-3p may be an important miRNA involved in the improvement of early SHR cardiac function via blood flow-restricted resistance training.

## 5. Conclusion

Among the differentially expressed miRNAs identified by RNA-seq and standardized analysis in the left ventricular myocardial tissue of early spontaneous hypertensive rats adapted to 8 weeks of blood flow-restricted resistance training, miR-224-5p, miR-31a-5p, miR-200b-3p, miR-200a-3p, miR-342-3p, miR-429, miR-1949, miR-1249, and miR-350 may be new targets involved in the mechanism of action of blood flow-restricted resistance training in improving hypertensive cardiac function, among which miR-200b-3p has the most significant differential expression.

## Supporting information

S1 Table Reverse transcription mixture ratios. (DOCX)

S2 Table Amplification procedures. (DOCX)

S3 Table Primer sequences. (DOCX)

S4 Table Reaction system mixture ratio. (DOCX)

S5 Table Reaction procedures. (DOCX)

S6 Table The miRNA target genes. (XLS)
