# Supplementary material for: Blood flow-restricted resistance training modulates miRNAs to improve early hypertensive cardiac function
Source: PLoS One. 2025 Sep 25;20(9):e0333027. doi: 10.1371/journal.pone.0333027 (PMC12463276; doi:10.1371/journal.pone.0333027)
Supplement: S1 Table — (DOCX) [file pone.0333027.s001.docx]

**S1 Table. Reverse transcription mixture ratios.**

| **Reagent** | **Amount used** | **Final concentration** |
| --- | --- | --- |
| **5X PrimeScript RT Master Mix（Perfect Real Time）** | 2 µl | 1X |
| **Total RNA** | 1 µl |  |
| **RNase Free dH2O** | up to 10 µl |  |
