# Supplementary material for: Blood flow-restricted resistance training modulates miRNAs to improve early hypertensive cardiac function
Source: PLoS One. 2025 Sep 25;20(9):e0333027. doi: 10.1371/journal.pone.0333027 (PMC12463276; doi:10.1371/journal.pone.0333027)
Supplement: S2 Table — (DOCX) [file pone.0333027.s002.docx]

**S2 Table. Amplification procedures.**

| **Procedure** | **Temperature** | **Time** |
| --- | --- | --- |
| **Reverse transcription reaction** | 37 ℃ | 1 h |
| **Reverse transcriptase inactivation reaction** | 85 ℃ | 5 min |
| **Maintaining temperature** | 4 ℃ |  |
