# Supplementary material for: Blood flow-restricted resistance training modulates miRNAs to improve early hypertensive cardiac function
Source: PLoS One. 2025 Sep 25;20(9):e0333027. doi: 10.1371/journal.pone.0333027 (PMC12463276; doi:10.1371/journal.pone.0333027)
Supplement: S3 Table — (DOCX) [file pone.0333027.s003.docx]

**S3 Table. Primer sequences.**

| **Gene Name** | **Primer sequence (5‘to3’)** |
| --- | --- |
| **miR-200a-3p (F)** | TAACACTGT CTGGTAACGATGT |
| **miR-200a-3p (R)** | CATCTTACCGGACAGTGCTGGA |
| **miR-200b-3p (F)** | GCGGCTAATACTGCCTGGTAA |
| **miR-200b-3p (R)** | GTGCAGGGTCCGAGGT |
| **miR-342-3p (F)** | GCCCGCTTCAAGTAATCCAGG |
| **miR-342-3p (R)** | GTGCAGGGTCCGAGGT |
| **miR-350 (F)** | TGCGGTTCACAAAGCCCATAGAG |
| **miR-350 (R)** | CCAGTGCAGGGTCCGAGGT |
| **miR-429 (F)** | ACGGGCTAATACTGTCTGGT |
| **miR-429 (R)** | GTGCAGGGTCCGAGGT |
| **miR-1249 (F)** | ATAATACGCCCTTCCCCCCCT |
| **miR-1249 (R)** | AGTGCGTGTCGTGGAGTCG |
| **miR-1949 (F)** | ACACTCCAGCTGGGTATACCAGGATGTCAGC |
| **miR-1949 (R)** | TGGTGTCGTGGAGTCG |
| **miR-31a-5p (F)** | GCGGAGGCAAGATGCTGGCA |
| **miR-31a-5p (R)** | ATCCAGTGCAGGGTCCGAGG |
| **miR-224-5p (F)** | ACAAGTCACTAGTGGTTCC |
| **miR-224-5p (R)** | CAGTGATGTTGCGGTCTG |
| **U6 (F)** | CTCGCTTCGGCAGCACA |
| **U6 (R)** | AACGCTTCACGAATTTGCGT |
