# Supplementary material for: Blood flow-restricted resistance training modulates miRNAs to improve early hypertensive cardiac function
Source: PLoS One. 2025 Sep 25;20(9):e0333027. doi: 10.1371/journal.pone.0333027 (PMC12463276; doi:10.1371/journal.pone.0333027)
Supplement: S4 Table — (DOCX) [file pone.0333027.s004.docx]

**S4 Table. Reaction system mixture ratio.**

| **Composition** | **Volume** |
| --- | --- |
| **ddH2O** | 9 µl |
| **TB Green Advantage Premix （2X）** | 12.5 µl |
| **ROX Dye （50X）** | 0.5 µl |
| **Forward Primer （10 µM）** | 0.5 µl |
| **Reverse Primer （10 µM）** | 0.5 µl |
| **cDNA** | 2.0 µl |
| **Total volume** | 25 µl |
