# Supplementary material for: Blood flow-restricted resistance training modulates miRNAs to improve early hypertensive cardiac function
Source: PLoS One. 2025 Sep 25;20(9):e0333027. doi: 10.1371/journal.pone.0333027 (PMC12463276; doi:10.1371/journal.pone.0333027)
Supplement: S5 Table — (DOCX) [file pone.0333027.s005.docx]

**S5 Table. Reaction Procedures.**

| **Steps** | **Temperature** | **Time** | **Remarks** |
| --- | --- | --- | --- |
| **Initial denaturation** | 95 ℃ | 10 s |  |
| **PCR (x 40)** | | | |
| **Denaturation** | 95 ℃ | 5 s |  |
| **Annealing** | 55 ℃ | 30 s |  |
| **Extension** | 72 ℃ | 30 s | Collection of fluorescent signals |
| **Dissociation curve** | 95 ℃ | 60 s |  |
|  | 55 ℃ | 30 s |  |
|  | 95 ℃ | 30 s | Collection of fluorescent signals |
